# Supplementary material for: Are carbon emissions trading and green financial instruments synergistic? -Comprehensive quantitative research based on content analysis
Source: PLoS One. 2024 Mar 7;19(3):e0298601. doi: 10.1371/journal.pone.0298601 (PMC10919601; doi:10.1371/journal.pone.0298601)
Supplement: S1 Data — (ZIP) [file pone.0298601.s001.zip › Data/地级市Z_F工作报告.zip!/╕ú╜¿/╧├├┼╩╨/└┤╫╘╣1⁄2╓┌║┼í╛┬φ┐╦╩2╛▌═°í┐_2017.docx]

各位代表:

　　现在，我代表市人民政府向大会报告工作，请予审议，并请市政协委员提出意见。

　　一、2016年和过去五年工作回顾

　　我们深入贯彻习近平总书记系列重要讲话精神和对福建、厦门工作的重要指示，在省委省政府和市委的坚强领导下，全力抓好稳增长、促改革、调结构、惠民生、防风险各项工作，较好完成全年目标任务。预计地区生产总值3737亿元，增长8%；固定资产投资2170亿元，增长15%；财政总收入1083.3亿元, 增长8.2%，其中，地方级财政收入647.9亿元，同口径增长8.6%；城乡居民人均可支配收入增幅与经济增长基本同步；居民消费价格上涨1.7%；年度节能减排任务全面完成。面对超强台风“莫兰蒂”的正面袭击，全市军民众志成城，在全省大力支持下，防抗措施有力有效，把灾害损失降到最低，灾后重建取得重大胜利。

　　一年来的主要工作和成效是:

　　（一）产业转型步伐加快

　　创新驱动能力增强。承担国家级科技项目380项，新培育国家级高新技术企业281家，规上高新技术产业增加值占规上工业增加值的59.2%。各类专利授权1.15万件，新增国家行业标准82项。新增重点实验室、工程技术研究中心等39家。全国首个科技领军人才创新创业基地加快建设，引进各类双创团队3700多个3.6万余人，新培育成长型中小微企业492家。成为国家自主创新示范区、海洋经济创新发展示范市。

　　先进制造业实力提升。同安、翔安高新技术产业基地建设进展顺利，火炬（翔安）产业区等平台集聚效应增强。启动一批智能制造试点示范工程，联芯一期、电气硝子一期等项目建成投产，清华紫光、ABB工业中心等项目建设加快。电子、机械两大支柱产业稳步增长，生物医药、海洋高新、新材料、节能环保等战略性新兴产业快速成长。规上工业企业利润总额增长36.7%。

　　现代服务业快速发展。被确立为国家四大国际航运中心之一，获批国家现代物流创新发展城市和智慧物流城市试点，港口集装箱吞吐量增长4.5%。获评最具创新力国际会展城市和国家旅游休闲示范城市，会展业总体经济效益增长14.8%；接待国内外游客6760万人次，总收入965亿元，分别增长12%和16%。金融机构本外币存、贷款余额分别增长10.3%和13.9%。新增境内外上市公司5家、新三板挂牌企业88家、两岸股权交易中心挂牌企业近300家。软件信息、文化创意、服务外包、网络零售等产业大幅增长。

　　都市现代农业提质增效。农业产业集群实现销售收入670亿元，增长8.9%。同安入选国家农村产业融合发展试点示范区。深入推进新型城镇化，结合美丽乡村建设多渠道增加农民收入，新建“菜篮子”基地14个、设施农业项目17个、市级休闲农业示范点8家，有力促进农业增效、农民增收、农村繁荣。

　　（二）发展动力更加强劲

　　深化改革取得实效。全面推进营改增，制定实施35条降成本措施，累计为企业减负近200亿元。深入推进“放管服”改革，承接上级下放审批事项240项，对应取消55项，下放审批和公共服务事项216项，实现市、区、镇（街）三级权责清单全覆盖。加快商事登记制度改革，率先实施“五证合一”，新增各类商事主体9.7万户，增长23.6%。获评社会信用体系建设示范市。加快国企公司制股份制改革，市属国企资产、营业收入分别增长25.5%和13.6%。改革投融资体制，设立产业资金池，签约落地新材料、新能源等4支产业子基金，总规模达123.7亿元。

　　自贸试验区成效显著。推动投资贸易便利化，国际贸易“单一窗口”获评全国自贸试验区最佳实践案例，常态化开展关检“三互合作”和一站式查验，船舶通关效率提高50%以上。推动产业发展高端化，航空维修业产值增长23%，引进金融、类金融和投资公司3400家，跨境电商货值增长40%，文化保税等新兴业态加快发展。

　　对外开放步伐加快。认真落实国家“一带一路”倡议，生成一批互联互通、经贸合作项目。合同利用外资增长81.8%，新批境外投资额55.4亿美元，增长1.5倍，其中对“海丝”沿线国家投资增长1.3倍。中欧（厦门）班列开行超过百列，并通过海铁联运延伸至台湾。厦航荣获中国质量奖，增开直飞温哥华、墨尔本等洲际航线。实际利用台资增长30.8%。成功举办G20财政和央行副手会议、世界城市日论坛、两岸企业家峰会等重要会议，投洽会、南洋文化节、国际海洋周、国际时尚周等活动影响力进一步增强。

　　（三）城市发展水平提升

　　基础设施更加完善。集美新城、环东海域东部新城、翔安南部新城、马銮湾新城建设加快，完成投资820亿元。策划生成 “四个领域”补短板项目550个，总投资5280亿元，完成投资697亿元，竣工及在建项目占53%。地铁1、2、3号线累计完成25个车站主体结构，区间掘进48.4公里，6号线马銮湾段开建。翔安南路二期等建成投用。建成厦鼓航道二期工程，邮轮母港海上世界项目动工建设。总投资86.6亿元的抽水蓄能电站开工，一批水厂和水源联通工程建设加快。建成综合管廊33.8公里、海绵城市10.5平方公里。新一代气象雷达投用，宽带中国试点城市建设全面完成。荣获全国人防先进城市“三连冠”。

　　城市管理更加精细。推动交通、政务、社区、城管等领域数字化应用。整治交通拥堵，鹭江道等路段交通状况得到初步改善。出台网约车管理政策，增开38条公交线路、2条BRT线路，增辟路外公共停车位6071个，获评全国绿色交通城市。拆除“两违”917.8万平方米。完成63个老旧小区改造。

　　城乡环境更加优化。空气质量优良率98.9%。岛外小流域综合整治加快，238个自然村完成生活污水分散式治理，97个自然村纳入市政污水系统。实施13个积涝点和排水设施改造。新增园林绿地487公顷，造林绿化524公顷。海沧（西部）垃圾焚烧发电厂二期等工程加快推进。获批国家蓝色海湾整治行动试点城市，成为国家生态市。

　　（四）社会民生持续改善

　　社会事业繁荣发展。新改扩建中小学项目58个，开工建设公办幼儿园22所，新增学位2.6万个。分级诊疗“厦门模式”推向全国，家庭医生签约率22.7%。医高专升级为厦门医学院。鼓浪屿整治提升和申遗工作取得阶段性成果。一批文艺精品获省级以上奖项。我市运动员在里约奥运会获得2金1银1铜、打破2项世界纪录，在残奥会获得1银。成功举办国际马拉松、世界铁人三项等赛事。山海协作、对口帮扶工作扎实推进，妇女、儿童、残疾人、老龄、老体协、关心下一代工作不断加强，民族团结宗教和睦，海防、档案、方志工作全面进步。

　　社会保障力度加大。新增就业17万人，城镇登记失业率控制在4%以内。对5万人实行灵活就业社保补助，为2.7万家企业发放稳岗补贴。基本养老、基本医疗、工伤、失业、生育五项保险参保人数较快增长。企业退休人员月平均养老金提高到3291元。城乡低保标准提高到每人每月610元。爱心护理院扩建工程、爱鹭老年养护中心建设加快。实现居家养老服务全覆盖，获批国家医养结合试点城市。开工建设保障性住房8154套，建成4538套。开工建设公租房8129套，完成配租727人。出台限购、限贷、限价等调控措施，房价上涨过快势头得到初步遏制。

　　社会治理取得实效。顺利通过第二批全国社区治理和服务创新验收，社区社会组织增长42%。完善高危行业安全生产责任保险制度，强化交通、建筑、燃气等重点领域安全整治。创建全国食品安全示范城市，加强生产经营全过程监管。完善社会治安立体防控体系，刑事案件发案率、群体性事件分别下降11.3%和36.6%。

　　（五）政府自身建设不断加强

　　深入开展“两学一做”学习教育，严格执行中央八项规定精神和国务院“约法三章”，完成公务用车改革，全市“三公”经费支出减少8.2%，各类文件、会议减少10%，一批违纪违法案件得到严肃查处。扎实推进依法行政，加强重点领域立法，废止和宣布失效市政府文件202件，主动公开政府信息 3.4万件。政府与法院、检察院、工会互动联系持续加强。自觉接受人大法律监督、工作监督和政协民主监督，认真办理市人大代表建议275件、市政协提案431件，办复率均为100%，满意率分别为98.9%和95.3%。

　　各位代表！本届政府履职五年来，牢牢把握中央支持福建加快发展的重大历史机遇，积极应对经济下行压力，主动适应和引领经济发展新常态，持续加快跨岛发展，全力建设美丽厦门，经济社会发展取得明显进步。

　　——经济实力稳步提升。地区生产总值年均增长9.2%，财政总收入、地方级财政收入年均分别增长11%和12%，固定资产投资累计完成8320亿元，年均增长14.1%。三次产业结构从1.0∶51.1∶47.9调整为0.7∶41.4∶57.9。平板显示、金融服务、旅游会展、航运物流、软件信息5条产业链产值均突破千亿。高新技术企业突破1200家。现代服务业成为主导产业，在国家综合试点城市复评中获得优秀。

　　——中心城市作用增强。岛内外规划、基础设施建设、基本公共服务一体化进程加快，完成22个厦漳泉龙区域同城化项目。福厦、龙厦、厦深铁路和厦漳跨海大桥、厦安高速等重大工程建成通车，轨道交通、翔安机场、城市快速路网等交通体系加快推进。厦漳港口完成一体化整合，集装箱吞吐量居全球第16位。空港开通国内外航线212条，跻身全球百强机场。

　　——改革开放纵深推进。完成新一轮政府机构改革，市级行政权力事项精简52%，前置审批、中介服务事项分别清理规范47.9%和39.5%，名列中国服务型政府十佳城市。自贸试验区33项改革为全国首创，“多规合一”改革成为可复制推广样板，国际一流营商环境加速形成。实际利用外资97.4亿美元，25家境外世界500强企业在厦投资或增资扩产，外贸综合竞争力居全国百强城市第5位。

　　——对台交流更加紧密。两岸交流合作综合配套改革先行先试108项创新政策，“一区三中心”建设加快。实际利用台资17.8亿美元，一批重大项目落地。对台进出口贸易额348亿美元。厦金“小三通”旅客近800万人次，社区、教育、文化、医疗等领域对接交流深入推进，海峡论坛、台交会、文博会、图交会等40多项活动影响力不断扩大。

　　——生态文明深化建设。获评国家森林城市、海洋生态文明示范区。饮用水源地水质达标率100%，城市生活垃圾无害化处理率100%，城镇污水集中处理率94%。划定981平方公里生态控制线，城市建成区绿化覆盖率40.8%，人均公园绿地11.5平方米。修复岸线30公里，清淤1.7亿立方米，退垦还海8.6平方公里。万元生产总值综合能耗、二氧化碳排放量和主要污染物排放优于全国水平，空气质量稳居全国前列。

　　——民生福祉持续增进。城乡居民人均可支配收入年均分别增长9%和10%。增加就业92万人，转移农村劳动力9万人。义务教育发展基本均衡区获国家认定，大病医保实现城乡全覆盖，城乡低保标准实现全市统一，基本建成全民社保城市，基本形成多层次住房保障体系，成为国家公共文化服务体系示范区。社区治理创新模式向全国推广，连续三届获评全国社会治安综治优秀城市，荣获全国文明城市“四连冠”和双拥模范城“九连冠”。

　　成绩来之不易，这是在市委正确领导下，全市人民齐心协力、团结奋斗的结果。我谨代表市人民政府，向全市人民，向全体人大代表和政协委员、各民主党派、各人民团体和各界人士，向中央和省驻厦单位、驻厦部队、武警官兵，向关心支持厦门发展的台港澳同胞、海外乡亲和国际友人，表示衷心的感谢！

　　我们清醒地认识到面临的困难和挑战、存在的差距和不足，主要是:经济下行压力较大，地区生产总值、固定资产投资、规上工业增加值、社会消费品零售总额、外贸进出口总额等指标未能达到年初预期目标；二产偏小、三产偏慢，龙头企业不多、带动力不足，工业投资比例偏低，发展后劲有待增强；岛内外发展仍不平衡，岛外新城建设、产城融合需大力推进；教育、医疗、交通等民生保障需进一步改善，房价上涨过快；推进“放管服”改革、转变政府职能还未完全到位；有些干部担当意识、责任意识不强，办事效率不高，个别领导干部违纪违法造成不良影响。对此，我们要坚持问题导向，创新举措认真加以解决。

　　二、今后五年工作思路和2017年工作建议

　　今后五年，政府工作总体要求是: 高举中国特色社会主义伟大旗帜，以邓小平理论、“三个代表”重要思想、科学发展观为指导，深入贯彻习近平总书记系列重要讲话精神和对福建、厦门工作的重要指示，紧紧围绕“五位一体”总体布局和“四个全面”战略布局，不断强化“四个意识”，牢固树立和贯彻新发展理念，深入实施美丽厦门战略规划，坚持稳中求进工作总基调，坚持以提高发展质量和效益为中心，以推进供给侧结构性改革为主线，以实施创新驱动发展战略为动力，以增进人民福祉为根本，以加快跨岛发展为抓手，以落实管党治党责任为保障，不断破解发展难题、厚植发展优势，建设“五大发展”示范市，率先全面建成小康社会，为“再上新台阶、建设新福建”，为实现中华民族伟大复兴的中国梦作出新的更大贡献。

　　主要目标和任务是:全面落实市第十二次党代会各项决策部署，确保完成“十三五”规划目标，实现地区生产总值年均增长8.5%左右，人均生产总值和人类发展指数达到中等发达国家水平。

　　一是推动创新发展再上新台阶。建设国家自主创新示范区，主动融入全球创新体系，发展壮大新产业新业态新技术新模式，充分发挥企业主体作用，加速科技成果转化，建成具有较强国际影响力的区域科技创新中心。打造现代产业发展新高地，深入实施千亿产业链(群)培育工程，促进制造业与服务业深度融合，大力培育新的产业增长支柱，打造海峡西岸强大的高端制造业高地和最具竞争力的现代服务业集聚区。扎实推进供给侧结构性改革，深入实施“降成本、优环境”专项行动，切实扩大有效投资，加强新型消费和优质服务供给，加快国资国企市场化改革，支持民营经济发展壮大，积极发展混合所有制经济。加快建设质量强市和知识产权强市，构建产业发展、节能减排、社会管理和公共服务等领域的厦门标准体系，推进知识产权综合管理改革试点。建立创新驱动发展的体制机制，构建城市创新管理服务体系，力争到2020年全社会科技研发投入占生产总值比重达到4%；深入实施人才强市战略，打造更具吸引力和竞争力的人才特区。

　　二是推动协调发展再上新台阶。加快推进跨岛发展，科学统筹生产生活生态布局，拓展优化“一岛一带多中心”城市空间格局，扎实推进以人为核心的新型城镇化，全面提升岛内外一体化水平。增强城市发展的系统性协调性，建成总里程265公里的6条地铁线，建成翔安机场，基本建成“两环八射”快速路网和新城、产业基地主路网，畅通港口集疏运，完善覆盖城乡的市政设施、信息化基础设施体系。提升文明城市品牌效应，构建向上向善、和谐健康的城市文化，完善社会诚信体系，提升城市软实力；巩固双拥共建成果，建设军政军民团结的标杆城市。

　　三是推动绿色发展再上新台阶。加强环境保护和生态建设，落实主体功能区战略和生态控制线制度，加强生态整体保护、系统修复、综合治理，保护生态系统平衡和生物多样性；高标准建设海绵城市，新建一批社区公园和步行道，建成一批城市“绿肺”和生态湿地，提高饮用水质量。推动绿色低碳发展，建设低碳城市，提升生态工业园区，发展绿色产业、绿色交通、绿色建筑，倡导绿色生活方式，建成国家生态文明建设示范市，人居环境质量保持全国领先。

　　四是推动开放发展再上新台阶。打造国际一流营商环境，推进“放管服”改革，建立覆盖企业全生命周期、高效便捷的服务体系，力争在市场准入、行政服务、市场监管体系上达到国际先进水平，在公共安全、公共服务、文化融合等方面形成与国际接轨的体制机制。打造自贸试验区升级版，加快建设集贸易、投资、金融、科创的开放与创新为一体的综合改革区，加快建设开放型经济体系的风险压力测试区，加快建设提升政府治理能力的职能再造区，努力构筑对接“一带一路”和企业“走出去”的桥头堡。打造“一带一路”支点城市，加快建设海陆空交通枢纽、经贸合作枢纽、人文交流枢纽。深化综合配套改革，打造两岸融合发展示范区，使厦门成为台胞创业就业最活跃、居住生活最舒心的城市。

　　五是推动共享发展再上新台阶。构建城乡一体全国领先的公共服务体系，促进基础教育公平、优质、均衡发展，新增中小学学位10万个，重视发展继续教育，推动职业教育、高等教育与地方经济更紧密融合；建设健康厦门，建立公平可及、系统连续的分级诊疗体系和更高质量的医疗服务体系；推进文化惠民，打造文化产业发展先进城市；全面发展群众体育和竞技体育。构筑更加公平可持续的社会保障网，突出抓好底线民生，着力解决中低收入家庭的住房困难问题，扩大基本养老、基本医疗、工伤等保险覆盖面；严格落实安全生产责任制，确保食品药品安全；全面建设法治厦门、平安厦门，建设最具安全感的城市，努力实现社会治理体系和治理能力现代化。

　　各位代表！2017年，金砖厦门会晤将隆重举办，做好今年工作的意义特别重大。我们要全面贯彻落实中央和全省经济工作会议精神，坚持宏观政策要稳、产业政策要准、微观政策要活、改革政策要实、社会政策要托底的政策思路，按照市委的决策部署，扎实工作、奋力拼搏，以优异成绩迎接党的十九大胜利召开。

　　今年经济社会发展主要预期目标是:地区生产总值增长8.6%左右，固定资产投资增长16%，财政总收入增长7.5%，社会消费品零售总额增长11%，城乡居民人均可支配收入均增长8.5%，居民消费价格涨幅控制在3%左右，完成节能减排任务。

　　重点做好八个方面工作:

　　（一）坚持创新驱动发展，着力发展壮大实体经济

　　大力提升自主创新能力。实施国家自主创新示范区85项试点政策，加快104项支撑项目和84项平台项目建设。支持军民融合协同创新研究院、厦大石墨烯研究院等研发机构发展，鼓励骨干企业与高校组建创新战略联盟。培育科技“小巨人”企业，新增国家级高新技术企业100家以上。加快建设小微企业双创基地城市示范、科技与金融结合试点城市，壮大种子、天使、风投基金，拓展科技担保、投贷联动等新业务，建立有利于大众创业、万众创新的全方位服务体系。加强高层次人才引进，为各类人才来厦工作生活创造更好的环境。

　　重点发展高端制造业。紧盯产业链关键和缺失环节，实施一批补链、强链、扩链关键项目，提升产业效益链和价值链。积极引导高端产业向高新技术产业基地集聚。加快实施集成电路产业发展规划，设立500亿元产业基金，重点跟踪75个项目，打造国家级基地。加快发展生物医药、新材料、海洋高新等重点产业，大力培育龙头骨干和高成长性企业。力促盈趣科技等19个项目开工，加快三安集成电路等47个项目建设，力争IOI棕榈油加工等30个项目竣工，实现工业投资470亿元。编制实施制造业与互联网融合发展规划，培育物联网、大数据应用等新业态，支持企业应用数控技术和智能装备，引导资源要素向高新项目、高效产能集聚。

　　加快发展现代服务业。完善丙洲、美峰现代服务业基地配套，深化现代服务业综合改革试点，加快京东分拣中心、海峡收藏品交易中心等24个项目建设。大力发展软件信息服务业，提升软件园三期产业集聚效应。加快建设国际航运中心，发展海铁联运、内支线中转、国际中转业务，构建航运金融、保险、代理等临港物流服务体系，力争集装箱吞吐量突破1000万标箱。鼓励开发多种模式的城市商业综合体，促进商贸、金融、旅游等集聚发展。积极拓展新兴业态，推动供应链管理、工业设计、仲裁调解等高品质发展。

　　积极发展都市现代农业。推进农业供给侧结构性改革，推动农村一二三产融合发展。优化农业种植养殖结构，建成15个设施农业项目，提高农业生产规模化、集约化水平。扶持农业龙头企业，以深加工提高农副产品附加值。打造特色小镇，发展休闲农业、休闲渔业等乡村旅游。实施美丽乡村建设和移民造福工程，改善农村生产生活环境。

　　（二）强化供需共同发力，着力保持经济中高速增长

　　推动供给提质增效。深入推进“去降补”，加快淘汰落后产能，培育精益求精的工匠精神，鼓励开展个性化定制、柔性化生产，增品种、提品质、创品牌，扩大有效供给和中高端供给。加快商业、办公房地产去库存，科学调控土地供给量，促进房地产市场平稳健康发展。完善金融监管体系，规范融资借贷行为，防范化解金融风险。围绕降低企业制度性交易、税费、社会保险费、生产要素等方面成本，出台一批政策。加快补齐经济社会发展各领域短板，不断增强发展后劲。强化市场主体地位，加强预期引导，增强企业家信心。加快建设社会信用体系，完善事中事后监管，健全诚信激励和失信联合惩戒机制，促进信用信息广泛有效应用。

　　培育新兴消费热点。扩大旅游文体、健康养老、教育培训等领域消费，加快汽车、家电等商品消费转型，鼓励信用消费、大众化消费、绿色消费，促进多点支撑的消费增长。加快发展电子商务、共同配送、连锁经营等现代流通方式，开展物流标准化试点。合理布局消费网点设施，加快建设地铁沿线商圈，新增一批民生商业设施，创造更加便利实惠的消费环境。

　　积极扩大有效投资。围绕国家产业政策、重大规划、重点投向策划生成项目，提高投资精准性。大力推进“五个一批”，加快“四个领域”补短板，完成产业发展项目120个、投资405亿元，基础设施项目115个、投资120亿元，社会事业项目15个、投资20亿元，民生事业项目80个、投资205亿元。加快273个重大、重点项目建设。做细前期工作，强化要素保障，完善征迁机制，尽快把意向投资转化为实际投资。加强项目调度和施工组织，实施挂图作战，强力推进工程建设。

　　力促外贸平稳增长。培育技术、标准、品牌、质量、服务新优势，提升传统优势产品竞争力，确保出口增幅高于全国平均水平。加快外贸转型升级基地建设，扶持外贸综合服务企业发展。大力发展服务贸易和服务外包，促进服务贸易与货物贸易良性互动。鼓励先进技术装备、关键零部件和能源资源进口，做强做大酒类、水果等进口商品集散中心和生产生活资料批发物流中心。

　　（三）全力加快跨岛发展，着力建设国家中心城市

　　加快岛外新城建设。高起点、高标准、高层次、高水平建设岛外新城，差异化、特色化推动产城融合。集美新城重点加快软件动漫、文化旅游等产业集聚，完善医院、学校、文体等公共服务配套，提升人居环境吸引力，打造新城建设样板。环东海域东部新城、翔安南部新城、马銮湾新城重点加快主干路网、供水供电、污水处理、绿化景观等设施建设，推动新城连片成势。

　　提升本岛功能品质。严控开发强度，节约集约用地，加强空间整合、功能疏解、内涵提升，优化业态布局，加快建设金融服务、商务营运、旅游会展、消费购物中心。结合风貌建筑和环境保护，加快老城区保护性开发和城中村改造，完成60个老旧小区改造项目，完善室外活动场所等公共服务设施，打造一批完整社区。

　　优化城市基础设施。确保地铁1号线试运营，全面加快2、3、4、6号线建设，基本完成23个车站主体结构。力争翔安机场主体工程开工，加快配套工程建设，同步建设临空产业园区。加快建设第二西通道、同翔大道等22个重要通道，全线贯通“两环八射”外环，开工建设第二东通道、福厦高铁，加快第三东通道等前期工作。全面完成综合管廊和海绵城市试点任务。确保莲花水库汛前下闸蓄水，实现长泰枋洋水利枢纽一期工程向厦门供水。新建同安西山水厂，扩建高殿、翔安、杏林水厂，完成岛内供水主干管网更新改造20公里。建成西三线天然气配套工程，新增老旧小区管道燃气用户1万户。投资20亿元，建成投运500千伏厦门变电站三期等一批电力设施。

　　提高规划管理水平。实施规划提升工程，加强岛内外规划衔接，深化城市设计、专项规划和控制性详规。实施城市治理工程，稳步推进城市管理执法体制改革，完善“智慧城管”综合平台。开展“交通整治年”活动，推进25项整治任务，提升道路畅通能力。继续大力整治“两违”，综合治理市容市貌。加强灾害监测预警，建设防灾减灾工程，增强城市抗灾能力。

　　（四）做好金砖厦门会晤保障，着力增强城市综合服务能力

　　以最高效率、最实作风、最好效果和节俭原则做好会晤服务保障，决不辜负党中央和习近平总书记的信任与重托。

　　提升景观环境。深入实施灾后重建工程包，以“三线四片”重点区域为核心，打造一批园林绿化、夜景亮化、立面美化精品工程。改造提升9条道路市政设施和19条道路园林绿化，新增园林绿地400公顷，尽早恢复城市往日的优美和整洁。

　　提升城市文明。围绕弘扬社会主义核心价值观，把会晤保障与文明创建有机结合，开展市民素质提升行动，营造积极向上浓厚氛围，加快创建全国公共文明行为典范城市。开展主题外宣和民间外宣，向世界展示开放厦门、文明厦门的良好形象。

　　提升国际化水平。加强与金砖国家经贸文化交流，深化国际友城往来合作，打造国际交流重要城市。加快建设全域旅游示范市，培育引进一批国际会展项目和主体，提升旅游会展服务能力和保障水平。对照国际标准，完善道路交通、公共场所、景区景点等各类标识，打造一批国际社区和国际街区，提升城市知名度和影响力。

　　（五）不断深化改革开放，着力增强发展动力活力

　　强化重点领域改革。纵深推进“放管服”改革，对涉及事项逐一建档立卡，每季度向社会公布。深化商事制度改革，加快实行“五证合一”、证照分离，全面推行“双随机一公开”监管。深化国企改革，大力发展混合所有制经济，加强风险管理和内控体系建设。深化投融资体制改革，壮大产业引导基金和股权投资基金，推广PPP模式。强化预算执行管理和动态监控，开展专项资金使用绩效评估。对接中央税制改革，深化营改增。推进农村土地三权分置，完成土地承包经营权确权登记颁证。推进公立医院综合改革，全面开展控费，完善分级诊疗配套政策，家庭医生签约服务覆盖30%以上户籍人口、60%以上重点人群。

　　强化自贸区示范带动。深化体制机制、政策、产业、风险防控四大领域改革，继续推出一批创新举措。大力推进投资自由化，扩大商贸服务、社会服务等领域开放。创新贸易监管，试点特殊监管区外的航空保税维修，对接服务国家大飞机战略，建设世界知名航空维修基地。做大飞机租赁业务，加快拓展到船舶、医疗器械、大型设备等领域，打造融资租赁集聚区。推进跨境直邮等高端服务业发展，做大跨境电子商务产业园，建设文化艺术品保税交易平台。扩大跨境双向融资、资金池和人民币双向发债等业务，推动金融开放创新。

　　强化“一带一路”合作。在基础设施、投资贸易、旅游会展、人文交流等重点领域,策划生成一批重大平台和项目。做强做大中欧（厦门）班列，打造国家物流新通道。推动设立“海丝”投资基金、知识产权运营投资基金，积极对接亚投行和丝路基金，扩大项目合作。加快中国-东盟海洋合作中心建设。吸引有实力的企业在厦设立“走出去”区域总部。

　　强化招商引资实效。坚持引资引技引智并举，加强定位、定向、定点精准招商，拓展产业链招商、委托招商、以商引商，加大对世界500强、台湾百强、大型央企和重点民企的招引力度。对重大龙头项目，主要领导要紧盯不放、全力以赴；对意向项目，相关领导要挂钩负责、分头跟进。

　　强化区域开放协作。加快厦漳泉龙同城化，滚动更新合作项目。深化闽西南五市、闽粤赣十三市等区域合作，做好对口支援新疆、西藏、甘肃、宁夏、重庆等地工作。

　　（六）深化对台交流合作，着力促进两岸深度融合

　　推进产业对接。加快建设“一区三中心”，深化两岸新兴产业和现代服务业交流合作。大力推动集成电路、智能制造、生物医药、文化创意等优势产业融合发展。支持两地企业联合建设科研平台，推动技术和创新成果转移转化。继续引进台资金融机构，做大跨海峡人民币代理清算群。拓展两岸海空客货运直航、海上滚装、海运快件等业务，实施台湾输大陆商品快速验放机制。开发厦金澎“一程多站”旅游产品，加快建设海峡旅游服务中心。

　　推进交流交往。努力提升海峡论坛、两岸民间艺术节等交流活动实效。办好两岸社区治理、婚姻家庭等论坛，为基层民众搭建更多交流合作平台。加快建设两岸青创基地，吸引更多台湾青年来厦学习实习、就业创业。

　　（七）持续巩固生态优势，着力创建生态文明先行示范区

　　加强环境治理。打好大气、水、土壤污染防治“三大战役”，整治工业废气、机动车排气、扬尘污染、油烟污染，加快建设绿色海港、绿色空港；实行“河长制”，实施最严格的水资源管理，加快岛外九条小流域综合整治；对污染地块和潜在污染地块实行清单管理，防治固废污染。加强陆海统筹，控制排海污染物总量，防控海漂垃圾污染，加快海域清淤，推进五缘湾、马銮湾、杏林湾生态修复工程。

　　加大节能减排。严格项目准入，重点发展资源消耗低、环境污染少的产业，推广绿色清洁生产。开工建设绿色建筑、实施公共建筑节能改造各200万平方米。实现黄标车100%淘汰，推广新能源汽车及装备，加快建设公交场站和自行车道。加快东部固废中心建设。完成餐厨垃圾资源化利用和无害化处理试点任务。岛内全面推行垃圾分类和垃圾不落地，岛外扩大试点、逐步推开。

　　健全生态制度。聚焦体制机制创新，完成生态文明建设目标评价考核办法等10项年度改革任务。进一步完善生态文明建设和环境保护目标责任制、行政问责制，落实领导干部生态环境损害责任终身追究制。实行能源和水资源消耗、建设用地等总量与强度双控制度，探索建立碳排放权、节能量交易、生态环境损害赔偿等制度。严格环境执法，加快形成政府、企业、公众多元共治的环境治理体系。

　　（八）加大公共服务供给，着力提高人民群众获得感

　　促进教育事业均衡发展。加大公共教育投入，更多向岛外、农村倾斜，改善薄弱学校办学条件。实施学前教育新三年行动计划，开建幼儿园项目22个、增加学位6500个，积极缓解“入园难”问题。新改扩建中小学项目60个、增加学位2万个。加快外籍人员子女学校和普通高中国际化试点建设，满足多元就学需求。新建2所中职学校，不断提升现代职业教育水平。抓紧筹建国科大厦门微电子工程学院，确保明年正式招生。推进一流大学、一流学科建设，提升高校教学水平和创新能力，推动教育更好服务产业发展。

　　促进卫生事业提质发展。采取更加有效的措施解决“看病难、看病贵”，政府加大投入，减轻参保人员医治大病罕见病的费用负担，将部分特殊药品纳入补充医疗保险基金支付范围。建立中、重度失能人员长期护理保险制度。增加优质医疗资源供给，确保复旦大学中山厦门医院建成投用，加快翔安医院、马銮湾医院、环东海域医院等一批重大项目建设，加强基层卫生服务体系建设和能力提升。推进国家健康医疗大数据中心和产业园建设。优化妇幼保健，提高出生人口素质。

　　促进文体事业繁荣发展。深化公共文化服务体系标准化、基层综合文化中心两项全国试点建设。加快发展创意设计、影视传媒等文化产业，加强文化市场监管。繁荣发展哲学社会科学，普及科学知识。实施文化惠民工程，办好“书香活动月”，推动全民阅读。持续做好鼓浪屿申遗工作。新建、改造一批体育场馆，推动文体设施向公众开放。引进培育一批国际品牌赛事，推进体育产业规模化。

　　促进就业创业蓬勃发展。加强就业创业服务和就业岗位开发，落实降低社会保险费、就业补贴、企业稳岗补贴等政策，新增城镇就业16.5万人。完善创业平台功能，推动创业带动就业。加强农村劳动力转产转业和高校毕业生技能培训，提高就业能力。落实企业工资指导机制，加强劳动监察和争议仲裁，构建和谐劳动关系。

　　促进社会保障稳步发展。开工建设保障性住房1.5万套，完善周边配套，健全管理机制。及时落实各群体待遇调整提高政策。加强社保基金监管，确保安全和保值增值。健全城乡社会救助体系，城乡低保对象、特困人员救助供养标准分别提高6%和10%，开展巨灾保险，农村居民因灾倒房保额由1.6万元提高到5万元，让困难群众遇急有助、遇困有帮。建设21个农村幸福院和3个社区老年人日间照料中心。加快推动妇女、儿童、慈善、残疾人事业健康发展。

　　促进社会治理和谐发展。明确社会治安综治责任清单，切实履行主体责任和监管责任。推进城市公共安全管理，创新矛盾纠纷排查调处机制，健全风险防控与应急处置体系，增强城市抗风险能力。加强安全生产监管,坚决遏制重特大事故发生。全力保障食品安全，在全省范围内精选生态条件最好的地方建立一批优质安全的食品生产供应基地，健全可追溯体系，让市民群众吃得安全、吃得放心。提升双拥共建水平，争创国家军民融合创新示范区。

　　三、努力建设人民满意政府

　　牢固树立“四个意识”，坚决维护以习近平同志为核心的党中央权威，更加扎实地把省委、省政府和市委各项决策部署落到实处。

　　全面推进依法行政。加快建设法治政府，把政府活动全面纳入法治轨道，自觉运用法治思维和法治方式推动工作。自觉接受人大及其常委会法律监督、工作监督和政协民主监督。自觉接受社会监督和舆论监督，加强行政监察和审计监督，让权力在阳光下运行。

　　强化工作机制创新。在每个工作领域建立健全可量化、可操作、可考核、可追究的机制办法，实行精准管理、定量管理、从严管理。健全政府决策机制，充分听取各方面意见，让决策更加科学，经得起历史检验。健全责任落实机制，锲而不舍、全力以赴解难题，让每项政策、每个项目都落地见效。健全工作激励机制，鼓励创新、宽容失败，让广大干部愿干事、敢干事、干成事。健全督查问责机制，消除工作落实的堵点与盲区，不断提高政府执行力。

　　持续优化发展环境。对标国际一流，坚持问题导向，补齐施工许可、纳税、获得信贷等方面短板，加快形成法治化、国际化、便利化营商环境。切实转变工作作风，大力推进效能建设，推行“马上就办”，构建“亲”“清”新型政商关系，真正让企业和市民省时省力省钱省心。

　　切实做到廉洁奉公。坚定不移推进全面从严治党，履行主体责任，突出“五抓五看”，把党风廉洁建设落实到位。把纪律和规矩挺在前面，认真执行准则条例，严格落实中央八项规定精神。坚持有腐必惩、有贪必肃，严肃查处各类违纪违法案件，努力实现干部清正、政府清廉、政治清明。

　　各位代表！蓝图已绘就，征程已开启。让我们紧密团结在以习近平同志为核心的党中央周围，在市委的坚强领导下，开拓进取、真抓实干，为建设“五大发展”示范市而努力奋斗!
